# Endoscopic Appearance After Transpancreatic Precut Sphincterotomy: Classification and Implications for Biliary Cannulation

**DOI:** 10.3390/jcm15176890

**Published:** 2026-09-05

**Authors:** Takeshi Hisa, Shigeru Nishiyama, Shogo Sakata, Akiharu Kudo, Yui Ito, Aki Ego, Naoki Miyasaka, Nao Takeuchi, Takahiro Yamada, Shozo Osera, Hideki Fukushima

**Affiliations:** Department of Gastroenterology, Saku Central Hospital Advanced Care Center, Nagano 385-0051, Japan

**Keywords:** endoscopic retrograde cholangiopancreatography, pancreatic guidewire placement, pancreatitis, papillary morphology, pancreaticobiliary anatomy

## Abstract

**Background/Objectives:** Biliary cannulation remains a major challenge during endoscopic retrograde cholangiopancreatography (ERCP). Transpancreatic precut sphincterotomy (TPS) is an effective rescue technique, yet its technical endpoints and optimal post-TPS cannulation strategies have not been standardized. We aimed to establish a practical cannulation strategy based on post-TPS endoscopic appearance. **Methods:** We retrospectively analyzed 135 patients who underwent TPS for difficult biliary cannulation between 2008 and 2026. Endoscopic images and videos obtained before and after TPS were reviewed. Post-TPS appearance was evaluated by focusing on the bile duct structure and incised red epithelial surface, and clinical outcomes, adverse events, and predictors of successful biliary cannulation were assessed. **Results:** Post-TPS endoscopic appearances were classified into four patterns. A hypothesis-generating interpretation was performed to link these patterns to three previously described types of distal pancreaticobiliary anatomies—short common channel, long common channel, and separate orifices. Specifically, (1) a white duct-like structure outside the incised red epithelial surface indicated a short common channel; (2) a hole-like structure within the incised red epithelial surface and (3) the absence of any identifiable ductal structure indicated a long common channel; and (4) preservation of an alternative orifice was considered indicative of separate orifices. Selective biliary cannulation during the same session was successful in 74.1% of patients (overall success rate, 88.1%). Post-ERCP pancreatitis occurred in 12.6% of patients, and bleeding occurred in two cases. Procedures performed predominantly by non-experts showed higher initial success rates without increased adverse events compared with those performed by experts. Exploratory multivariable analyses revealed that adequate incision was the only significant predictor of successful biliary cannulation. **Conclusions:** Post-TPS endoscopic appearance may suggest the distal pancreaticobiliary anatomy and provide practical guidance for directing biliary cannulation.

## 1. Introduction

Difficult biliary cannulation is often encountered during endoscopic retrograde cholangiopancreatography (ERCP). Although transpancreatic precut sphincterotomy (TPS) is an effective and safe rescue technique in such situations [1,2], this technique has not yet been standardized, and the optimal strategy for achieving definitive biliary cannulation after TPS remains unclear. Recent observations suggest that Type 4 papillary morphology [3] may be a potential factor associated with difficult bile duct cannulation following TPS [4]. Therefore, in this study, we aimed to develop a practical cannulation strategy based on the endoscopic appearance of the papillae after TPS.

## 2. Materials and Methods

### 2.1. Study Design

This single-center retrospective study was conducted using the collected clinical data. The study protocol was approved by the ethics committee of the Saku Central Hospital Group (R201704-11).

### 2.2. Patients

We enrolled consecutive patients who underwent TPS for difficult biliary cannulation, which was defined, based on the European guidelines, as more than one unintended pancreatic duct cannulation [5]. Patients with a history of papillary intervention such as endoscopic sphincterotomy or endoscopic papillary balloon dilation and those with anatomical alterations other than distal gastrectomy with Billroth I reconstruction were excluded. Informed consent was obtained from all the patients or their proxies.

### 2.3. Procedures

Non-expert endoscopists performed the procedures under the supervision of an expert endoscopist (T.H.) who had performed more than 1000 ERCP procedures. Intravenous anesthesia consisted of midazolam and pentazocine as required, and flumazenil was administered to reverse the adverse effects of midazolam. Blood pressure, electrocardiogram, and oxygen saturation were monitored continuously. None of the patients were administered preprocedural rectal non-steroidal anti-inflammatory drugs (NSAIDs) or protease inhibitors.

Selective biliary cannulation was attempted using a duodenoscope (JF 260V; Olympus Medical Systems, Tokyo, Japan) and a catheter (ERCP catheter; MTW Endoskopie Manufaktur, Wesel, Germany; PR 10Q 1; Olympus). The catheter was preloaded with a 0.025-inch guidewire (VisiGlide2, Olympus; RevoWave, Piolax Medical Devices, Yokohama, Japan), and a contrast-assisted technique was used.

After pancreatic guidewire placement, TPS was performed toward the 11 o’clock direction using a sphincterotome (CleverCut3 V, Olympus; Engetsu, Kaneka Medix, Kanagawa, Japan) with mixed current (Endocut mode, 120 W, Effect 3) generated by an electrosurgical unit (ICC200; Erbe Elektromedizin, Baden Württemberg, Tuebingen, Germany).

A pancreatic stent (Geenen; Cook Medical, Bloomington, IN, USA) was placed at the discretion of the operator. When biliary cannulation remained unsuccessful, a second ERCP, percutaneous transhepatic biliary drainage, or endoscopic ultrasound-guided biliary drainage was considered, based on the patient’s condition. Endoscopic images and videos were recorded both before and after TPS. Antithrombotic agents were administered in accordance with the guidelines of the Japanese Society of Gastroenterological Endoscopy [6,7].

### 2.4. Outcomes and Definitions

**Figure 1:**
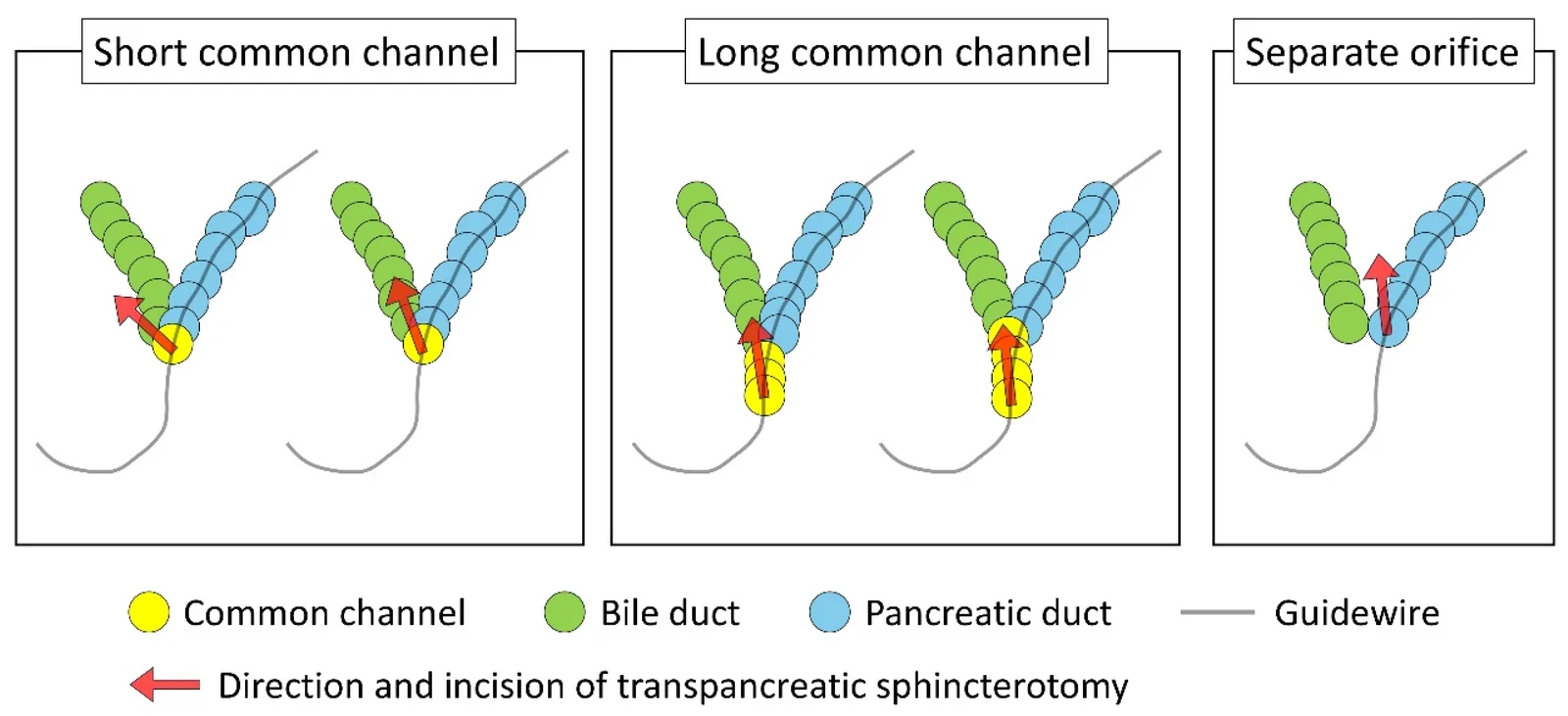
Schema of distal pancreaticobiliary duct anatomy. The distal pancreaticobiliary anatomy was classified as short common channel, long common channel, and separate orifices.

The effectiveness and safety of TPS were evaluated by assessing the biliary cannulation success rate and incidence of adverse events. The endoscopic appearance after TPS was classified according to cases with successful biliary cannulation. Distal pancreaticobiliary anatomy was categorized into three types: short common channels, long common channels, and separate orifices [8] (Figure 1). The expert endoscopist (T.H.) classified the post-TPS endoscopic appearance using endoscopic images and videos obtained during the procedure.

To evaluate reproducibility, two non-expert endoscopists (N.M. and A.E.) independently interpreted all post-TPS images using the same predefined criteria. Before image interpretation, they received a brief structured training session to ensure consistent application of the classification. The expert endoscopist (T.H.) was not blinded to clinical information because the study was retrospective and the classification system was newly developed. Anatomical categories were assigned retrospectively by matching the post-TPS endoscopic appearance to the most plausible distal pancreaticobiliary anatomy.

The definitions and severity grading of post-procedural bleeding and pancreatitis were based on internationally recognized criteria [9,10], with minor modifications [11]. Post-procedural pancreatitis was defined as the occurrence of abdominal pain accompanied by raised serum amylase levels at least three times the upper limit of normal levels on the day following the procedure.

### 2.5. Statistical Analysis

Statistical analyses were performed using EZR (Easy R, version 4.5.2, Saitama Medical Center, Jichi Medical University, Saitama, Japan), which is a graphical user interface for R and R Commander. Nominal variables were compared using Pearson’s χ^2^ test or Fisher’s exact test, and continuous non-normally distributed variables were compared using the Wilcoxon rank-sum test. Statistical significance was defined as a two-sided *p*-value < 0.05.

Variables with *p* < 0.10 in the univariate analysis were included in the exploratory multivariable logistic regression models. When multiple variables met the *p* < 0.10 threshold, these were entered directly into the model. In the analysis where only one variable met this criterion, clinically important variables with relatively lower *p* values were additionally included. To avoid overfitting, the number of variables was restricted according to the event-per-variable principle, and a forced-entry method was applied. Multivariable analyses were performed to identify independent predictors while adjusting for potential confounders.

Interobserver agreement for the endoscopic classification was evaluated using Cohen’s κ statistic. The 95% confidence intervals (CIs) for κ were calculated using the standard error derived from the asymptotic variance, and κ values were interpreted according to the criteria proposed by Landis and Koch. Percent agreement was also reported to complement κ values because of their susceptibility to prevalence bias.

For proportion-based outcomes, including pattern-specific success rates, exact binomial 95% CIs were calculated using the Clopper–Pearson method.

## 3. Results

### 3.1. Patient Characteristics

**Between** September 2008 and May 2026, 6537 patients underwent ERCP, of whom 135 underwent TPS. The patient characteristics are summarized in Table 1. The median age was 80.5 years, and 68 patients were male. The indications for ERCP included bile duct stones (*n* = 62) and carcinoma (*n* = 61), including pancreatic carcinoma (*n* = 19). Sphincter of Oddi dysfunction was observed in six patients, and acute cholecystitis and post-operative bile leakage were observed in three patients each.

Regarding papillary morphology [3], types 1, 2, 3, and 4 were observed in 45, 14, 33, and 43 patients, respectively. In total, 88 procedures were performed by non-expert endoscopists and 47 procedures by expert endoscopists. A pancreatic stent was placed in 36 (26.7%) patients.

### 3.2. Effectiveness and Safety of TPS

The effectiveness and safety outcomes of the TPS are summarized in Table 2. Selective biliary cannulation after TPS was successful in 100 (74.1%) patients during the same session. A second ERCP was performed in 24 patients, and biliary cannulation was achieved in 19 patients, resulting in a final success rate of 88.1%.

Post-ERCP pancreatitis (PEP) occurred in 17 patients (12.6%), including 16 mild cases and one severe case. Post-procedural bleeding occurred in two patients; one patient undergoing hemodialysis developed bleeding on day 6 post-TPS and was treated with electrical coagulation, and the other patient, who was receiving antiplatelet therapy, developed bleeding on day 2 and underwent hemostasis with a hypertonic saline–epinephrine injection. No perforations were observed in any of the patients.

To evaluate the learning curve, all patients were divided into two equal groups, and the outcomes were compared between the early and late periods (Table 3). During the early period (2008–2020), 54.4% of the procedures were performed by expert endoscopists, with initial and final biliary access rates of 66.2% and 82.4%, respectively. In contrast, during the late period (2021–2026), procedures were performed predominantly by non-expert endoscopists (85.1%, *p* < 0.001), and both the initial and final biliary access rates were significantly higher (82.1% and 94.0%, respectively; *p* < 0.05). Notably, there were no significant differences in the adverse events between the two periods.

### 3.3. Classification of Post-TPS Endoscopic Appearance

Overall, sufficient incision could not be achieved in 29 patients. “Insufficient incision” represented technical failure, indicating that TPS was not adequately achieved in these patients; therefore, they were excluded from further classification. Among the remaining 106 patients, the post-TPS endoscopic appearance was classified into four patterns based on the bile duct structure and the incised red epithelial surface (Table 2).

**Figure 2:**
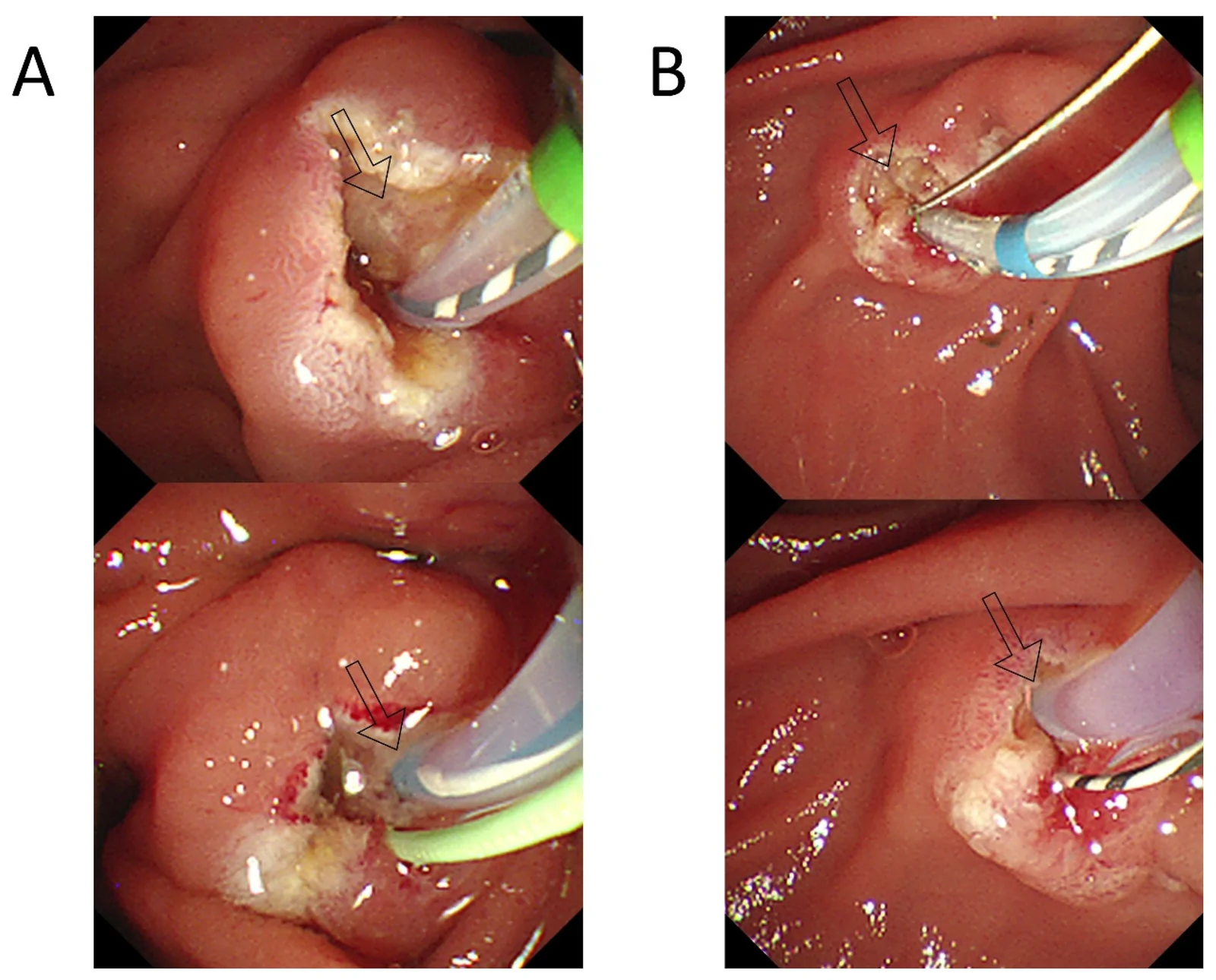
Post-TPS endoscopic appearance (short common channel). (**A**) In the upper panel, a white cord-like structure (arrow) is observed outside the incised red epithelial surface. In the lower panel, a catheter is inserted into the bile duct, which appears as a white cord-like structure. (**B**) In the upper panel, a white bamboo-split-like structure (arrow) is observed outside the incised red epithelial surface. In the lower panel, a catheter is inserted into the bile duct, which appears as a white, bamboo-split-like structure. TPS, transpancreatic precut sphincterotomy.

**Figure 3:**
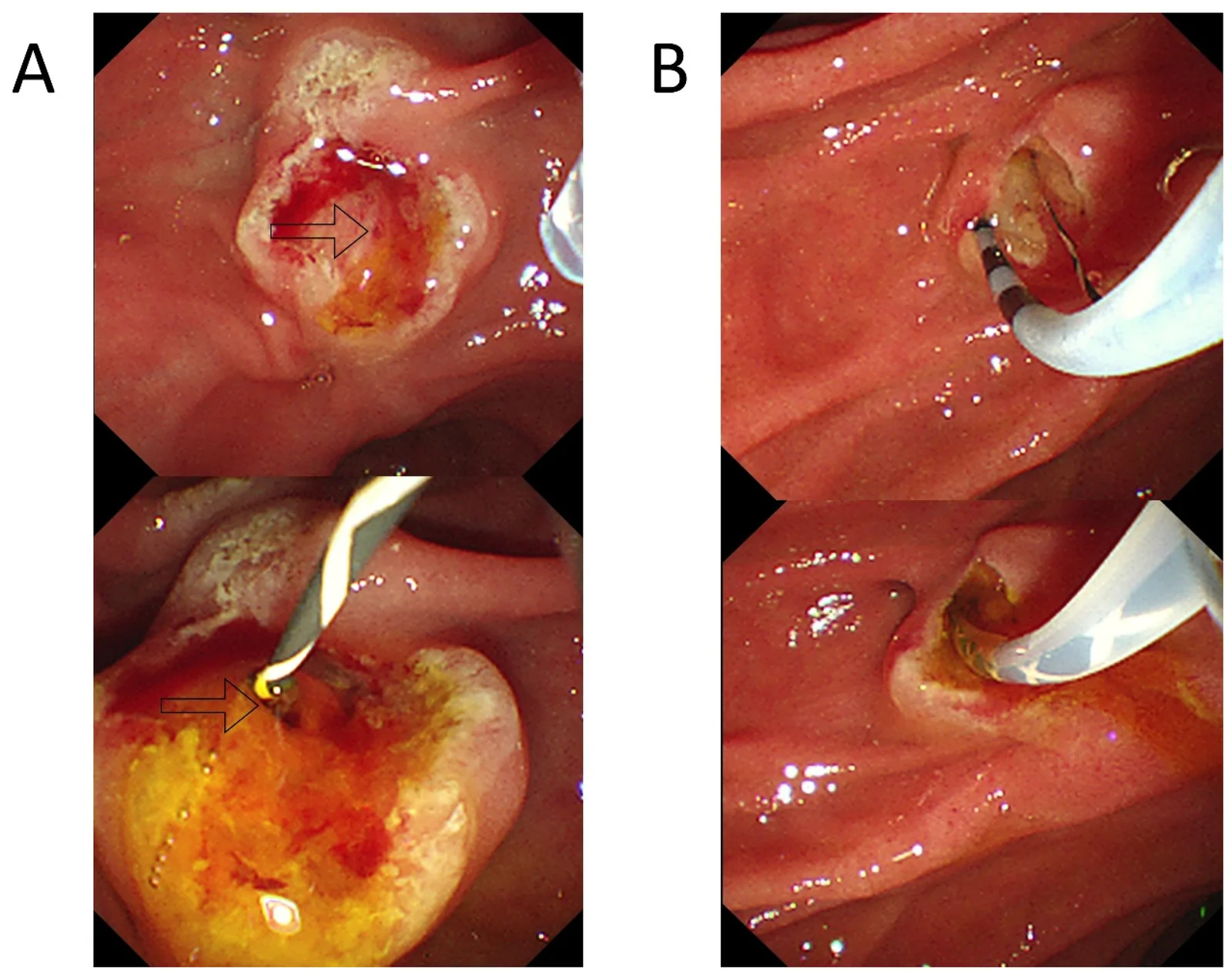
Post-TPS endoscopic appearance (long common channel). (**A**) In the upper panel, a hole-like structure (arrow) is observed within the incised red epithelial surface. In the lower panel, a guidewire is inserted into the bile duct, which appears as a hole-like structure. (**B**) In the upper panel, no duct-like structure is observed either within or outside the incised red epithelial surface. In the lower panel, a catheter is inserted into the deep bile duct along a pancreatic guidewire. TPS, transpancreatic precut sphincterotomy.

**Figure 4:**
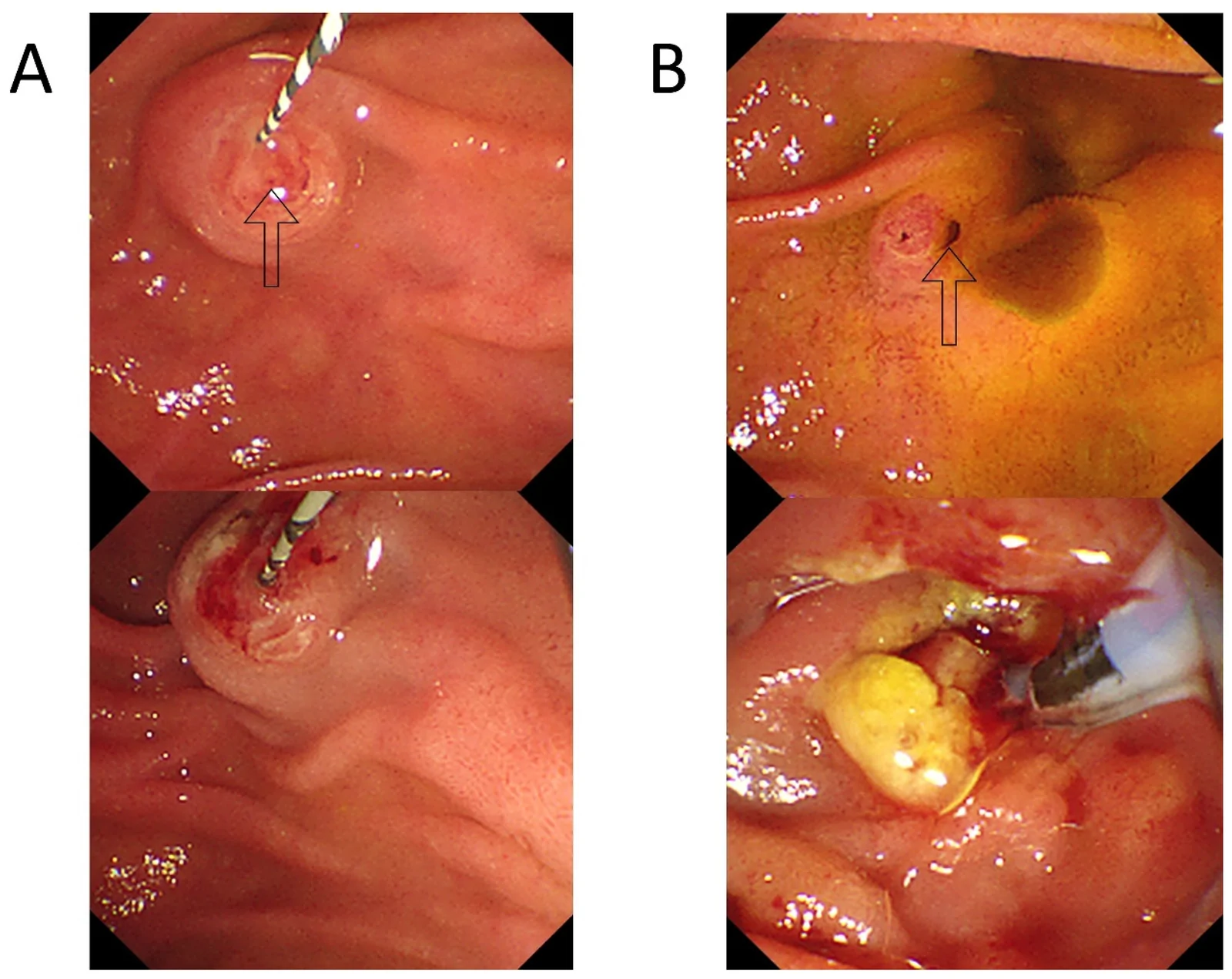
Post-TPS endoscopic appearance (separate orifices). (**A**) In the upper panel, a guidewire is deployed into the pancreatic duct, and a pinhole-like orifice (arrow) is visible at the center of the papilla. In the lower panel, the pinhole like orifice remains after TPS, and a guidewire is inserted into the bile duct, which appears as a pinhole like orifice. (**B**) In the upper panel, left and right orifices are observed, with bile flowing from the right orifice (arrow). In the lower panel, the intact right orifice remains after TPS is performed from the left orifice, and a catheter is inserted into the bile duct, which appears as the intact right orifice. TPS, transpancreatic precut sphincterotomy.

1. A white duct-like structure outside the incised red epithelial surface (Figure 2).
2. Hole-like structures within the incised red epithelial surface (Figure 3A).
3. No ductal structure other than the incised red epithelial surface (Figure 3B).
4. An intact alternative orifice (Figure 4).

Patterns 1, 2, 3, and 4 were observed in 53, 2, 47, and 4 patients, respectively.

**Figure 5:**
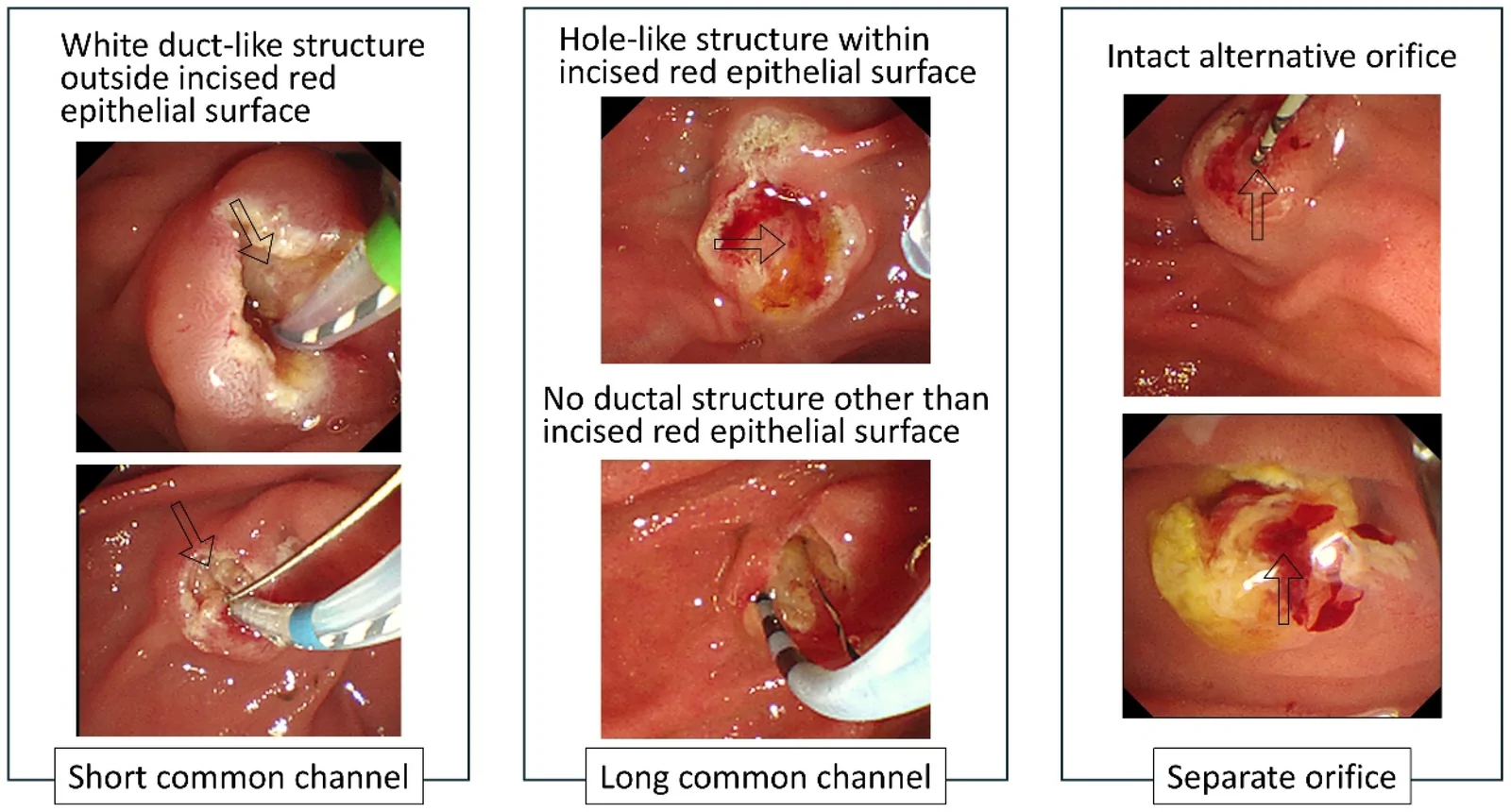
Distal pancreaticobiliary anatomy inferred from post-TPS endoscopic appearance. When a white duct-like structure is observed outside the incised red epithelial surface after TPS, the anatomy corresponds to that of a short common channel. When a hole-like structure is observed within the incised red epithelial surface or when no duct-like structure is identified, the anatomy corresponds to that of a long common channel. When an intact alternative orifice is observed after TPS, the anatomy corresponds to that of separate orifices. TPS, transpancreatic precut sphincterotomy.

As a hypothesis-generating interpretation linking these endoscopic appearance patterns with previously described anatomical patterns, we evaluated their relationship with the distal pancreaticobiliary anatomy. As shown in Figure 5, Pattern (1) corresponded to a short common channel, Patterns (2) and (3) corresponded to a long common channel, and Pattern (4) corresponded to separate orifices.

Regarding the relationship between the distal pancreaticobiliary anatomy, as inferred from post-TPS endoscopic appearance, and the papillary morphology (Table 4), among the 53 patients with short common channels, papillary morphology Types 1, 2, 3, and 4 were observed in 18, 6, 10, and 19 patients, respectively. Among the 49 patients with long common channels, Types 1, 2, 3, and 4 were observed in 8, 6, 16, and 19 patients, respectively; among the four patients with separate orifices, Types 1, 2, 3, and 4 were observed in 3, 1, 0, and 0 patients, respectively. When papillary morphology was dichotomized, Type 3 showed a higher proportion of long common channels compared with non-Type 3 (61.5% vs. 41.3%), although the difference did not reach statistical significance (χ^2^ = 3.26, *p* = 0.07).

Regarding post-TPS endoscopic appearance, the biliary access rates were 100% for Pattern 1 (53/53), Pattern 2 (2/2), and Pattern 4 (4/4), with corresponding exact 95% confidence intervals of 93.3–100%, 15.8–100%, and 39.8–100%, respectively. Pattern 3 showed a biliary access rate of 91.5% (43/47), with an exact 95% confidence interval of 79.6–97.6%. Regarding distal pancreaticobiliary anatomy, the biliary access rates were 100% for the short common channel (53/53) and separate orifice (4/4) groups, with corresponding exact 95% confidence intervals of 93.3–100% and 39.8–100%, respectively. A biliary access rate of 91.8% was achieved for patients in the long common channel group (45/49), with an exact 95% confidence interval of 80.4–97.7%. In contrast, cases categorized as “insufficient incision” demonstrated a markedly lower biliary access rate of 58.6% (17/29), with an exact 95% confidence interval of 38.9–76.5%.

### 3.4. Predictors for Successful Same-Session Biliary Cannulation After TPS and Post-TPS Pancreatitis

For successful same-session biliary cannulation after TPS, sufficient incision of TPS remained the only independent predictor in both univariate and exploratory multivariable analyses (Table 5). In contrast, for post-TPS pancreatitis, age ≥ 65 years was the only factor that remained significant in both analyses (Table 6).

### 3.5. Interobserver Agreement in Classification of Post-TPS Endoscopic Appearance

Interobserver agreement for the four-pattern endoscopic classification was assessed among three endoscopists: one expert endoscopist (T.H.), who developed the classification, and two non-expert endoscopists with experience of fewer than 70 ERCP procedures (N.M. and A.E.). The reported κ values were 0.59 (95% CI, 0.48–0.70) for T.H.–N.M., 0.63 (95% CI, 0.52–0.74) for T.H.–A.E., and 0.49 (95% CI, 0.37–0.61) for N.M.–A.E., with corresponding percent agreements of 77.4%, 79.2%, and 71.7%, respectively (Table 7).

## 4. Discussion

In this study, we classified the post-TPS endoscopic appearance according to the distal pancreaticobiliary anatomy, focusing on the bile duct structure and incised red epithelial surface. In patients with a short common channel, a white duct-like structure was observed outside the incised red epithelial surface. In patients with a long common channel, the ductal structure appeared either as a hole-like opening within the incised red epithelial surface or was not identifiable at all. In patients with a separate orifice, an intact alternative orifice was observed. However, a separate orifice could often be identified during precannulation assessment, which may help avoid unnecessary TPS. Noteworthily, these patterns of the distal pancreaticobiliary anatomy were not confirmed by cholangiographic delineation of the common channel, bile duct, or pancreatic duct, nor by existing reference standards such as endoscopic ultrasonography, magnetic resonance cholangiopancreatography, or surgical findings. Therefore, we propose them as hypothesis-generating interpretations that link post-TPS endoscopic appearances with previously described anatomical patterns.

The interobserver agreement for the four-pattern classification showed moderate-to-substantial κ values, indicating reasonable reproducibility even among non-expert endoscopists. Although κ values were affected by pattern prevalence, differences in experience, early-stage interpretive ambiguity, and the known limitations of κ in imbalanced datasets, the overall percent agreement remained high (71–79%), supporting the practical applicability of the classification. Further refinement of pattern definitions and structured training for non-experts may improve reproducibility.

Although the endpoint of TPS is the exposure of the bile duct [12], the ductal structure may not be visible within the incised red epithelial surface in cases with a very long common channel. Additionally, several conditions that necessitate TPS make achieving an adequate incision difficult. Given that perforation after TPS has been reported in 1.9% [12] and 11.5% [13] of cases, the incision should be limited to the minimum required extent. In our study, cases categorized as “insufficient incision” were associated with a markedly lower success rate of 58.6%, indicating that most failed cannulation cases were associated with inadequate TPS incision. Furthermore, exploratory multivariable analyses revealed incision adequacy to be the only significant predictor of successful biliary cannulation after TPS. Taken together, these findings indicate that successful biliary cannulation with TPS critically depends on performing an adequate incision, rather than on the underlying papillary morphology [4]. In clinical practice, the presence of a ductal structure should first be explored either outside or within the incised red epithelial surface within the feasible extent of the incision. If no ductal structure is identified, deep cannulation along the pancreatic guidewire, considering the possibility of a long common channel, may facilitate biliary access. These appearance-based cues may assist endoscopists in selecting reasonable cannulation approaches; however, their potential impact on procedural safety should be interpreted with caution.

Given that the success rate of biliary cannulation using the TPS varies from 53.8% [13] to 95.9% [14], the same-session success rate of 74.1% achieved in this study is consistent with previously reported outcomes. A recent systematic review and network meta-analysis comparing various techniques, including conventional cannulation, precut papillotomy, precut fistulotomy, delayed precut papillotomy, delayed precut fistulotomy, TPS, and the double-guidewire technique, demonstrated that TPS was the only method associated with superior cannulation efficacy [2]. However, as TPS is generally performed with a pancreatic guidewire in place, concerns regarding the risk of PEP remain. In the largest multicenter randomized controlled trial to date [12], the PEP rate after TPS was 13.5% (14/104), despite NSAID administration in 78.8% patients and pancreatic stent placement in 8.7% patients.

In our study, PEP occurred in 12.6% of patients; notably, none received NSAIDs, and prophylactic pancreatic stent placement was discretionary. Consequently, guideline-recommended prophylactic measures [5,15]—including routine rectal NSAIDs and prophylactic pancreatic duct stenting, in both universal and high-risk-selected approaches—were not applied. Exploratory multivariable analyses identified only age ≥ 65 years as an independent predictor of post-TPS pancreatitis, and no conclusions can be drawn regarding the impact of pancreatic-stent absence. Because our study spans an 18-year period during which ERCP devices, PEP prevention strategies, and indications for therapeutic ERCP changed substantially, adherence to contemporary guideline-based preventive strategies might have further reduced PEP incidence. These temporal changes and the partial implementation of guideline-recommended prophylactic measures represent limitations of our study.

Although the long-term course after TPS remains unclear, a previous study with more than 4 years of follow-up (median, 6 years) reported no significant difference in post-TPS late adverse event occurrence compared with those in a control group [16]. Although the European Society of Gastroenterological Endoscopy guidelines recommend that TPS be performed by expert endoscopists [5], a recent study found no significant differences in biliary cannulation success or adverse event occurrence between procedures performed by non-expert and expert operators [17]. In our study, despite the higher proportion of non-expert endoscopists in the late period, the biliary access rate was significantly higher, without an increase in adverse events. However, these improvements cannot be attributed solely to the increased participation of non-expert endoscopists; temporal factors—such as advances in equipment, accumulated institutional experience, supervision, and evolving clinical practice—may also have influenced the outcomes.

Because blind cannulation after TPS can induce bleeding and edema, thereby exacerbating the difficulty in biliary access, a systematic strategy based on endoscopic findings may help guide cannulation attempts, even for non-expert endoscopists. The proposed post-TPS classification offers a structured framework for interpreting the endoscopic appearance after TPS, and may assist non-expert endoscopists in selecting more appropriate cannulation approaches rather than relying on blind attempts. Accordingly, we believe that our classification provides incremental value by linking visual endoscopic findings to concrete decision-making, although its impact on procedural expertise requires further validation. Any potential influence on procedural safety or efficiency, however, warrants careful consideration.

This study had some limitations. First, this was a single-center retrospective study. However, it included one of the largest patient cohorts reported for TPS. Second, the proposed patterns of distal pancreaticobiliary anatomy were not validated by imaging examinations; therefore, these represent hypothesis-generating interpretations that link post-TPS endoscopic appearances with previously described anatomical patterns. Future studies are warranted to validate our findings using imaging or cholangiographic assessments. Third, image evaluation was performed by an expert endoscopist (T.H.), who was not blinded to the clinical information. Because this was a retrospective study and used a newly developed classification system, blinding was not feasible. To assess reproducibility, two non-expert endoscopists independently interpreted all images after receiving structured training. Although some subjectivity may be unavoidable, consistent criteria were applied. Prospective validation under blinded conditions will be important.

## 5. Conclusions

Post-TPS endoscopic appearance may offer practical visual cues to the underlying distal pancreaticobiliary anatomy and help guide biliary cannulation. Recognizing these appearance patterns can support more consistent and efficient cannulation strategies. Adequate incision remains the key determinant of successful biliary access. Prospective studies with blinded assessment and imaging confirmation are needed to validate and refine this appearance-based classification.

## Figures and Tables

**Table 1:** Baseline patient characteristics.

Total patients, *n*	135	
Median age, y [IQR]	80.5 [72–86.5]	(range: 43–100)
Male, *n*, %	68	50.4
Purpose of ERCP, *n*, %		
Bile duct stone	62	45.9
Carcinoma	61	45.2
(Pancreatic carcinoma)	19	
Sphincter of Oddi dysfunction	6	4.4
Acute cholecystitis	3	2.2
Postoperative bile leak	3	2.2
Papillary morphology *, *n*, %		
Type 1	45	33.3
Type 2	14	10.4
Type 3	33	24.4
Type 4	43	31.9
Operator, *n*, %		
Non-expert	88	65.2
Expert	47	34.8
NSAIDs use before ERCP, *n*, %	0	0
Pancreatic stent, *n*, %	36	26.7

IQR, interquartile range; *, refers to [3]; NSAIDs, nonsteroidal anti-inflammatory drugs; ERCP, endoscopic retrograde cholangiopancreatography.

**Table 2:** Outcomes of TPS.

**Biliary Access, *n*, %**		
Same-session success	100	74.1
Repeat ERCP	24	
Delayed success	19	
Final success	119	88.1
**Post-procedural Adverse Events, *n*, %**	19	14.1
Pancreatitis	17	12.6
Mild	16	
Severe	1	
Bleeding	2	1.5
Perforation	0	
**Post-TPS Endoscopic Appearance, *n***		
Ductal structure outside incised red epithelial surface	53	
Ductal structure within incised red epithelial surface	2	
No ductal structure outside/within incised red epithelial surface	47	
Intact alternative orifice	4	

TPS, transpancreatic precut sphincterotomy; ERCP, endoscopic retrograde cholangiopancreatography.

**Table 3:** Learning curve analysis of TPS: comparison between early and late period.

	Early Period (2008–2020)		Late Period (2021–2026)		*p*-Value
Patients, *n*, %	68	50.4	67	49.6	
Operator, *n*, %					<0.001
Expert	37	54.4	10	14.9	
Non-expert	31	45.6	57	85.1	
Biliary access, *n*, %					
Initial success	45	66.2	55	82.1	0.035
Final success	56	82.4	63	94.0	0.036
Adverse events, *n*, %	10	14.7	9	13.4	N.S.
Pancreatitis	8	11.8	9	13.4	N.S.
Bleeding	2	2.9	0	0	N.S.

TPS, transpancreatic precut sphincterotomy; N.S., not significant.

**Table 4:** Relationship between distal pancreaticobiliary anatomy inferred from post-TPS endoscopic appearance and papillary morphology.

	Papillary Morphology *			
Distal Pancreaticobiliary Anatomy	Type 1	Type 2	Type 3	Type 4
Short common channel (*n* = 53)	18	6	10	19
Long common channel (*n* = 49)	8	6	16	19
Separate orifice (*n* = 4)	3	1	0	0

TPS, transpancreatic precut sphincterotomy; *, refers to [3].

**Table 5:** Univariate and exploratory multivariable logistic regression analyses of predictors for successful same-session biliary cannulation after TPS.

	Univariate Logistic Regression		Exploratory Multivariable Logistic Regression	
Predictor	OR (95% CI)	*p*-Value	Adjusted OR (95% CI)	*p*-Value
Age < 65 years	0.86 (0.26–2.85)	0.809	—	—
Male	1.20 (0.59–2.78)	0.523	—	—
No periampullary diverticulum	1.26 (0.50–3.21)	0.624	—	—
Ampullary morphology type 1/2/3	0.55 (0.23–1.34)	0.188	0.82 (0.28–2.36)	0.711
Non-pancreatic-head cancer	0.34 (0.07–1.58)	0.171	0.27 (0.05–1.62)	0.153
Expert endoscopist	0.74 (0.33–1.64)	0.455	—	—
Sufficient incision of TPS	17.3 (6.41–46.4)	<0.0001	17.5 (6.28–49.0)	< 0.0001

TPS, transpancreatic precut sphincterotomy; OR, odds ratio; CI, confidence interval.

**Table 6:** Univariate and exploratory multivariable logistic regression analyses of predictors for post-TPS pancreatitis.

	Univariate Logistic Regression		Exploratory Multivariable Logistic Regression	
Predictor	OR (95% CI)	*p*-Value	Adjusted OR (95% CI)	*p*-Value
Age ≥ 65 years	3.68 (1.11–12.20)	0.034	4.10 (1.12–15.0)	0.033
Female	2.75 (0.91–8.29)	0.073	2.90 (0.92–9.10)	0.069
Periampullary diverticulum	0.84 (0.22–3.16)	0.795	—	—
Pancreatic duct stenosis	1.37 (0.35–5.29)	0.652	—	—
Endoscopic papillary balloon dilation	1.30 (0.26–6.43)	0.75	—	—
Non-expert endoscopist	0.73 (0.26–2.07)	0.557	—	—
Unsuccessful biliary cannulation	0.14 (0.02–1.11)	0.063	0.14 (0.02–1.08)	0.059
No pancreatic stent	NA	0.709	—	—

TPS, transpancreatic precut sphincterotomy; OR, odds ratio; CI, confidence interval, NA, not available/not applicable.

**Table 7:** Interobserver agreement for post-TPS endoscopic appearance.

Pair	% Agreement	κ Value	95% CI	Interpretation
T.H.–N.M.	77.4%	0.59	0.48–0.70	Moderate
T.H.–A.E.	79.2%	0.63	0.52–0.74	Substantial
N.M.–A.E.	71.7%	0.49	0.37–0.61	Moderate

TPS, transpancreatic precut sphincterotomy; CI, confidence interval.

## Data Availability

The data underlying this study are not publicly available due to patient privacy and institutional regulations. De-identified data may be made available from the corresponding author upon reasonable request and with approval from the institutional ethics committee.

## Abbreviations

The following abbreviations are used in this manuscript:

ERCP	endoscopic retrograde cholangiopancreatography
TPS	transpancreatic precut sphincterotomy
NSAIDs	non-steroidal anti-inflammatory drugs
PEP	post-endoscopic retrograde cholangiopancreatography pancreatitis
